# Protective Effects of Serotonin, L-Carnitine, and Coenzyme Q10 on Human Sperm Cryopreservation: Functional and In Silico Evidence

**DOI:** 10.3390/jcm15208134

**Published:** 2026-10-20

**Authors:** Yasemin Yilmazer, Aleyna Cavdar, Zeynep Aydin Sinirlioglu, Sinem Dogan, Turgut Aydin

**Affiliations:** 1 Department of Molecular Biology and Genetics, Istanbul Sabahattin Zaim University, Istanbul 34303, Türkiye; zeynep.sinirlioglu@izu.edu.tr; 2 Department of Bioengineering, Faculty of Chemical and Metallurgical Engineering, Yıldız Technical University, Istanbul 34210, Türkiye; aleyna.cavdar@kent.edu.tr; 3 Department of Biochemistry, Faculty of Pharmacy, Istanbul Kent University, Istanbul 34406, Türkiye; 4 ResearchKent, Istanbul Kent University, Istanbul 34406, Türkiye; 5 Department of Obstetrics and Gynecology, Acibadem Mehmet Ali Aydinlar University, Istanbul 34303, Türkiye; sinemercandogan@gmail.com (S.D.); turgut.aydin@acibadem.com (T.A.)

**Keywords:** sperm cryopreservation, cryopreservation media, serotonin, sperm DNA fragmentation, in silico analysis

## Abstract

**Background**: Sperm cryopreservation can impair sperm function through oxidative stress, membrane damage, mitochondrial dysfunction, and DNA fragmentation. This study evaluated the effects of supplementing a basal freezing medium with serotonin (5-HT), L-carnitine (L-C), and coenzyme Q10 (CoQ10) on post-thaw human sperm quality and explored potential molecular interactions using molecular docking. **Methods**: Semen samples from 63 normozoospermic volunteers were processed using the swim-up technique, and each sample was divided into five aliquots assigned to the following conditions: commercial freezing medium; basal freezing medium; basal medium supplemented with 5-HT alone; basal medium supplemented with L-C and CoQ10; and basal medium supplemented with all three compounds. Post-thaw motility, intracellular ROS, viability, and apoptosis-associated changes were assessed in all 63 donors; DNA fragmentation was assessed in a subset of 32 donors. Molecular docking explored predicted interactions of 5-HT, L-C, and coenzyme Q1 (CoQ1), a short-chain CoQ10 analogue, with selected sperm-related targets. **Results**: The combined formulation showed the highest median total (54%) and progressive (39%) motility, the lowest median percentage of DCF-positive spermatozoa (11.30%), and the lowest median DNA fragmentation index (2.58%). Pairwise analyses showed significant differences between the combined formulation and several comparator groups, depending on the outcome. Viability was significantly higher with the combined formulation than with basal, 5-HT-supplemented, and L-C/CoQ10-supplemented media (all Bonferroni-adjusted *p* < 0.001). Docking predicted interactions of 5-HT with HTR4 and SLC6A4 and of CoQ1 with CPT1A. **Conclusions**: Combined 5-HT, L-C, and CoQ10 supplementation was associated with favorable changes in several post-thaw sperm quality parameters. These findings support further investigation of this formulation, while the predicted molecular interactions require experimental validation.

## 1. Introduction

Sperm cryopreservation is an established approach for preserving male fertility and is particularly important for individuals who may undergo gonadotoxic treatments such as chemotherapy or radiotherapy, as well as surgical procedures that may compromise reproductive function. Cryopreserved sperm can subsequently be used in assisted reproductive technologies, including intrauterine insemination, in vitro fertilization, and intracytoplasmic sperm injection. The ultimate objective of sperm cryopreservation is to maintain sperm viability, motility, structural integrity, genomic stability, and fertilization potential during long-term storage [1,2,3,4,5].

Despite advances in cryopreservation techniques and freezing media, spermatozoa remain highly susceptible to cryopreservation-induced injury. The freeze–thaw process can disrupt plasma membrane integrity and lipid organization, impair mitochondrial function, alter acrosomal structure, reduce sperm viability and motility, and increase DNA fragmentation. These effects have been associated with osmotic stress, cold shock, intracellular ice formation, mitochondrial dysfunction, excessive production of reactive oxygen species, and disruption of endogenous antioxidant defenses [1]. Oxidative stress is particularly important because spermatozoa contain high levels of polyunsaturated fatty acids and have relatively limited antioxidant capacity, making them vulnerable to lipid peroxidation and oxidative damage to cellular and genomic components.

Serotonin (5-hydroxytryptamine, 5-HT) has emerged as a potential regulator of male reproductive function. Components of the serotonergic system have been identified in human spermatozoa, and serotonin has been implicated in the regulation of sperm motility and other sperm functions [6,7,8,9,10]. In vitro studies have reported increased linear and curvilinear sperm motility following serotonin exposure, while studies in other species have suggested roles for serotonin in sperm motility, fertilization, and the acrosome reaction [11,12]. Serotonergic signaling has also been associated with vas deferens contraction, testicular blood flow, testicular development, and testosterone production. The observation that epididymal serotonin levels change during sexual maturation further supports a potential role for serotonin in male reproductive physiology [13,14]. However, the potential protective role of serotonin during sperm cryopreservation remains insufficiently characterized.

L-carnitine (L-C) and coenzyme Q10 (CoQ10) are also of interest because of their complementary roles in cellular energy metabolism and oxidative defense. L-C facilitates mitochondrial fatty-acid transport and contributes to energy production, while its antioxidant properties may help protect spermatozoa from oxidative damage. Previous studies have shown that supplementation with L-C can improve post-thaw sperm motility, viability, and DNA integrity, supporting its potential use in sperm cryopreservation media [15]. CoQ10 is an essential component of the mitochondrial electron transport system and a lipid-soluble antioxidant. By supporting mitochondrial energy production and limiting oxidative damage, CoQ10 may contribute to the maintenance of sperm motility and protection of membrane lipids, mitochondria, and DNA. Seminal CoQ10 levels have been associated with sperm concentration and motility, and supplementation has been reported to improve several sperm quality parameters, particularly in men with impaired fertility [16,17,18]. Importantly, the complementary biological activities of 5-HT, L-C, and CoQ10 suggest that their combined use may provide broader protection against the multiple mechanisms involved in cryopreservation-induced sperm injury.

The potential mechanisms underlying the actions of these compounds can also be considered at the molecular level. HTR4 and SLC6A4 are key components of serotonergic signaling and may mediate the effects of serotonin on sperm function. CPT1A is involved in mitochondrial fatty-acid metabolism and is therefore relevant to the energy requirements of sperm motility. CATSPER1 is a sperm-specific calcium channel that contributes to calcium influx, hyperactivation, and flagellar function, whereas glutathione peroxidase 4 is an important antioxidant enzyme involved in protection against lipid peroxidation [19,20,21]. Together, these targets represent distinct biological processes relevant to sperm function, including serotonergic signaling, energy metabolism, calcium homeostasis, and antioxidant defense. Investigating the potential interactions of 5-HT, L-C, and CoQ10-related molecules with these targets may therefore provide exploratory insight into their potential molecular interactions.

Although 5-HT, L-C, and CoQ10 have individually been associated with sperm function and protection against oxidative damage, the effects of their combined supplementation in a sperm cryopreservation medium have not been sufficiently investigated. Furthermore, the molecular interactions that may contribute to the potential complementary actions of these compounds during cryopreservation remain unclear. Therefore, the present study evaluated whether supplementation of a basal freezing medium with 5-HT alone, L-C plus CoQ10, or all three compounds improves post-thaw human sperm quality. Total and progressive sperm motility, intracellular reactive oxygen species levels, sperm viability, apoptosis, and DNA fragmentation were evaluated following cryopreservation. In addition, molecular docking analyses were performed to investigate the potential interactions of 5-HT, L-C, and coenzyme Q1, a short-chain analogue of CoQ10, with selected sperm-related molecular targets, including HTR4, SLC6A4, CPT1A, CATSPER1, and glutathione peroxidase 4.

## 2. Materials and Methods

### 2.1. Participants

A total of 63 volunteers who attended the Infertility Clinic at Acıbadem Mehmet Ali Aydınlar University Atakent Hospital (Istanbul, Turkey) were included in this laboratory-based experimental study with a paired, within-donor design. The study was conducted between 2 September 2022, and 2 September 2024. The study protocol was reviewed and approved by the Acıbadem Mehmet Ali Aydınlar University Institutional Ethics Committee (Approval no. ATADEK-2022-14/30), and the study was conducted in accordance with the ethical standards of institutional and national research committees and the Declaration of Helsinki. Written informed consent was obtained from each participant prior to sample donation.

### 2.2. Sample Collection

Semen samples were collected via masturbation following 3–5 days of sexual abstinence in sterile, disposable Petri dishes and left to liquefy at 37 °C. Subsequently, semen volume, sperm concentration and motility were determined based on the World Health Organization guidelines for the examination and processing of human semen [22]. Sperm concentration and motility were determined using a Makler counting chamber under ×20 objective magnification after loading 10 µL of semen. Sperm motility analysis was performed for all 63 participants included in the study (*n* = 63). Only normozoospermic samples meeting the WHO reference values were included in the study. Semen samples were processed using the swim-up technique by carefully overlaying sperm wash medium onto the liquefied semen, followed by incubation at 37 °C for 30 min. The upper motile sperm fraction was collected and centrifuged at 500× *g* for 10 min. The supernatant was discarded and the sperm pellet was resuspended in Origio Sperm Wash Medium (Origio^®^, Malov, Denmark). Sperm concentration and motility were re-evaluated. Sperm motility was classified into three categories: progressively motile, non-progressively motile, and immotile, according to the WHO criteria.

Each washed sperm sample obtained from a single donor was equally divided into five aliquots so that every donor served as its own control. The aliquots were assigned to five experimental groups: Group 1, Origio sperm freezing medium (MED-10670010; Origio^®^, Malov, Denmark); Group 2, the Base Freezing Medium (BFM), which consisted of HAM F-10 supplemented with 10% human serum albumin (HSA), 10% glycerol, 50 mM sucrose, and 15 mM glycine; Group 3, BFM supplemented with serotonin (5-HT); Group 4, BFM supplemented with L-carnitine (L-C) and coenzyme Q10 (CoQ10); and Group 5, BFM supplemented with 5-HT, L-C, and CoQ10. Based on our preliminary optimization study, the optimal concentrations of serotonin (5-HT; sc-298707; Santa Cruz Biotechnology, Dallas, TX, USA), L-carnitine (L-C; SA-840092; SigmaAldrich, St. Louis, MO, USA), and coenzyme Q10 (CoQ10; SAC9538; Sigma-Aldrich) were determined to be 200 µM, 0.5 mg/mL, and 0.01 mg/mL, respectively, and these concentrations were used in all subsequent experiments [7]. 5-HT and L-carnitine were dissolved in water, whereas CoQ10 was dissolved in dimethyl sulfoxide (DMSO) prior to supplementation of the freezing medium. The final concentration of DMSO in the prepared medium was 0.2% (*v*/*v*).

Equal volumes of sperm suspension and the corresponding freezing medium were mixed gently (1:1, *v*/*v*) and transferred into sterile cryovials. The cryovials were placed in liquid nitrogen vapor approximately 10 cm above the liquid nitrogen surface for 20 min before being plunged into liquid nitrogen for long-term storage. Following cryopreservation, sperm samples were maintained in liquid nitrogen for 5–45 days. The variation in storage duration (5–45 days) reflected differences in sample collection dates and the scheduling of post-thaw laboratory analyses; all samples were maintained continuously under identical liquid nitrogen storage conditions until analysis. For thawing, cryovials were removed from liquid nitrogen, kept at room temperature for 1 min, and then immersed in a 37 °C water bath until completely thawed. The thawed suspension was washed with an equal volume of sperm washing medium, centrifuged at 500× *g* for 10 min, and the final sperm pellet was resuspended in sperm washing medium before further analyses. After thawing, sperm intracellular reactive oxygen species (ROS), apoptosis, and DNA fragmentation were evaluated.

### 2.3. Evaluation of ROS Levels in Human Spermatozoa

Intracellular ROS levels were determined by flow cytometry (CytoFLEX, Beckman Coulter Life Sciences, Brea, CA, USA) using 2′,7′-dichlorodihydrofluorescein diacetate (H2DCF-DA; Cat. No. D399, Invitrogen, Carlsbad, CA, USA), an oxidation-sensitive probe. ROS analysis was performed for all 63 participants included in the study (*n* = 63).

Following thawing, each sample was divided into an unstained control fraction and a test fraction, both adjusted to a concentration of 25 × 10^5^ spermatozoa/mL. The unstained control sample was analyzed without any probe and used to determine background autofluorescence. H2DCF-DA was added to the test fraction at a final concentration of 10 µM, and the suspension was incubated in the dark at 25 °C for 30 min prior to analysis. ROS levels were expressed as the percentage of DCF-positive spermatozoa relative to the unstained control.

### 2.4. Evaluation of Sperm Viability and Apoptosis-Associated Changes

Sperm viability and apoptosis-associated membrane changes were assessed using an Annexin V–fluorescein isothiocyanate (FITC) apoptosis detection kit (BioLegend, San Diego, CA, USA) combined with propidium iodide (PI; Vector Laboratories, Newark, CA, USA). While Annexin V–FITC labels phosphatidylserine exposed on the outer leaflet of the plasma membrane in apoptotic cells, PI enters only those cells that have lost membrane integrity. Post-thaw sperm viability and apoptosis analyses were performed for all 63 participants included in the study (*n* = 63).

Spermatozoa were adjusted to a concentration of 2 × 10^6^/mL, and 500 µL of the suspension was mixed with an equal volume of cell staining buffer. Annexin V–FITC (5 µL) and PI (5 µL) were added to each tube; the samples were then incubated at 25 °C in the dark for 15 min. Subsequently, Annexin V binding buffer (400 µL) was added, and the entire volume was analyzed using a CytoFLEX cytometer. The characteristic sperm population was identified by its pear-shaped distribution in the forward-scatter/side-scatter (FSC/SSC) plot. A sperm-specific gate was applied to minimize the inclusion of debris before Annexin V/PI quadrant analysis.

Within the gated sperm population, four populations were defined according to their Annexin V-FITC/PI staining patterns: Annexin V-negative/PI-negative (viable), Annexin V-positive/PI-negative (early apoptosis-associated), Annexin V-positive/PI-positive (late apoptosis-associated), and Annexin V-negative/PI-positive (necrotic), following previously reported staining-based classifications in human spermatozoa [23]. The percentage of each population was calculated relative to the total number of events within the sperm gate. These categories represent operational classifications based on staining patterns. Phosphatidylserine externalization in spermatozoa may also occur in association with membrane remodeling or cryopreservation-induced membrane alterations; therefore, Annexin V positivity was interpreted cautiously as an apoptosis-associated membrane change rather than definitive evidence of the classical apoptotic pathway [24].

### 2.5. Evaluation of Sperm DNA Fragmentation

DNA fragmentation was measured using a TUNEL-based method (In Situ Cell Death Detection Kit, Roche Diagnostics, Mannheim, Germany) in accordance with the manufacturer’s instructions. DNA fragmentation analysis was performed in 32 participants (*n* = 32) for whom sufficient sperm cells were available for the complete assay procedure. The remaining 31 donors were not included in the DNA fragmentation analysis because insufficient sperm cells were available to complete the TUNEL assay. These donors remained included in the motility, ROS, and Annexin V/PI analyses. Following thawing, samples were diluted in PBS to a concentration of 5 × 10^6^ cells/mL and centrifuged at 300× *g* for 10 min. The cell pellets were fixed at room temperature for 60 min, washed with PBS, and permeabilized on ice for 2 min using a 0.1% Triton X-100 solution prepared in 0.1% sodium citrate.

Following a washing step, the cells were incubated with the TUNEL reaction mixture (enzyme and label solutions) for 60 min at 37 °C in the dark; they were then washed once more with PBS and analyzed using a CytoFLEX flow cytometer (Beckman Coulter Life Sciences, Brea, CA, USA). At least 10,000 sperm events were recorded for each sample. The DNA fragmentation index (DFI) was expressed as the proportion of TUNEL-positive spermatozoa among all analyzed spermatozoa. To validate the analysis performance, negative and positive controls were processed concurrently in each run.

### 2.6. Molecular Docking Analysis

Targets were chosen to represent the three axes through which the compounds are proposed to act on sperm: serotonergic (HTR4, SLC6A4), calcium-dependent motility (CATSPER1) and mitochondrial fatty-acid oxidation (CPT1) [6,12,19]. GPX4 was included as a mechanistically distinct comparator [20]. Docking was performed against the serotonin transporter SLC6A4, the receptor HTR4, the enzymes CPT1A and GPX4, and the sperm-specific cation channel subunit CATSPER1. The structures of serotonin (PubChem CID: 5202), L-carnitine (PubChem CID: 10917) and the short-chain analogue coenzyme Q1 (PubChem CID: 4462) were obtained from the PubChem database [25]. CoQ1 was used as a surrogate for CoQ10 because the C50 polyprenyl tail of CoQ10 expands the conformational search space beyond the range in which rigid-receptor docking yields reliable poses, and dominates scoring through nonspecific hydrophobic contacts [26]. The structures of SLC6A4 (PDB ID: 6VRH), HTR4 (PDB ID: 7XT8) and GPX4 (PDB ID: 6HN3) were obtained from the Protein Data Bank, whereas CPT1A and CATSPER1, for which no full experimental structures were available, were retrieved from the AlphaFold Protein Structure Database (UniProt P50416 and Q8NEC5) [27]. The ligand-binding regions were centred on the co-crystallized ligand for SLC6A4 and HTR4 and on the catalytic residues for GPX4 (residues 46, 81, 136 and 137) and CPT1A (His473); for CATSPER1, where no defined pocket was available, blind docking centred on the protein was used. Grid box centres were x = 135.1, y = 123.7, z = 122.3 for SLC6A4; x = 99.4, y = 119.0, z = 88.2 for HTR4; x = −0.2, y = −4.0, z = −1.9 for CPT1A; x = −12.0, y = −4.5, z = 0.5 for CATSPER1; and x = −11.6, y = 2.9, z = −6.6 for GPX4, within a 22 Å box (30 Å for the CATSPER1 blind docking). Ligands were prepared with RDKit (ETKDGv3, MMFF94) and Meeko 0.7.1, and all protein preparation steps and docking procedures were performed using AutoDock Vina (version 1.2.7) with an exhaustiveness of 32 and three random seeds [28,29]. The Vina score was used to compare ligand–target interactions. Interactions were visualized in PyMOL (open-source version 3.2.0) [30]. Validation was limited to two internal checks: three independent random seeds per ligand–target pair, and GPX4 as a comparator. Redocking of co-crystallized ligands with RMSD assessment was not performed and was not possible for the two AlphaFold models. Predicted poses are therefore unvalidated at the atomic level, and residue-level contacts are provisional.

### 2.7. Exclusion Criteria

Men over the age of 40 were not eligible for the study. Candidates presenting with varicocele, testicular atrophy, ejaculatory disorders, Sertoli cell-only syndrome, or other endocrine or anatomical anomalies, including azoospermia, oligozoospermia, or asthenozoospermia, as well as samples with heavy bacterial contamination and samples containing more than 1 × 10^6^ peroxidase-positive leukocytes per milliliter, were not included in the study.

### 2.8. Statistical Analyses

All statistical analyses were carried out using GraphPad Prism (version 11.0.2; GraphPad Software, San Diego, CA, USA) and Python (version 3.13, with the SciPy and Statsmodels libraries). Data distribution was assessed using the Shapiro–Wilk normality test. Because each donor semen sample was split into five aliquots corresponding to the five experimental conditions, the data inherently comprise paired repeated measurements. Therefore, to account for within-subject dependencies, the non-parametric Friedman test was performed across all continuous parameters (total motility, progressive motility, intracellular ROS, sperm viability/apoptosis populations, and DNA fragmentation index). When significant omnibus differences were detected (*P* < 0.05), pairwise post-hoc comparisons were conducted using the Wilcoxon signed-rank test with Bonferroni correction to control the family-wise error rate. Data are presented as median with interquartile range (IQR), unless otherwise stated. A two-sided adjusted *P* value (*P_adj_* < 0.05) was considered statistically significant. Statistical significance was denoted as follows: ns, not significant (*P*_adj ≥ 0.05); *P*_adj < 0.05 (*); *P*_adj < 0.01 (**); *P*_adj < 0.001 (***).

## 3. Results

### 3.1. Post-Thaw Sperm Motility

**Figure 1:**
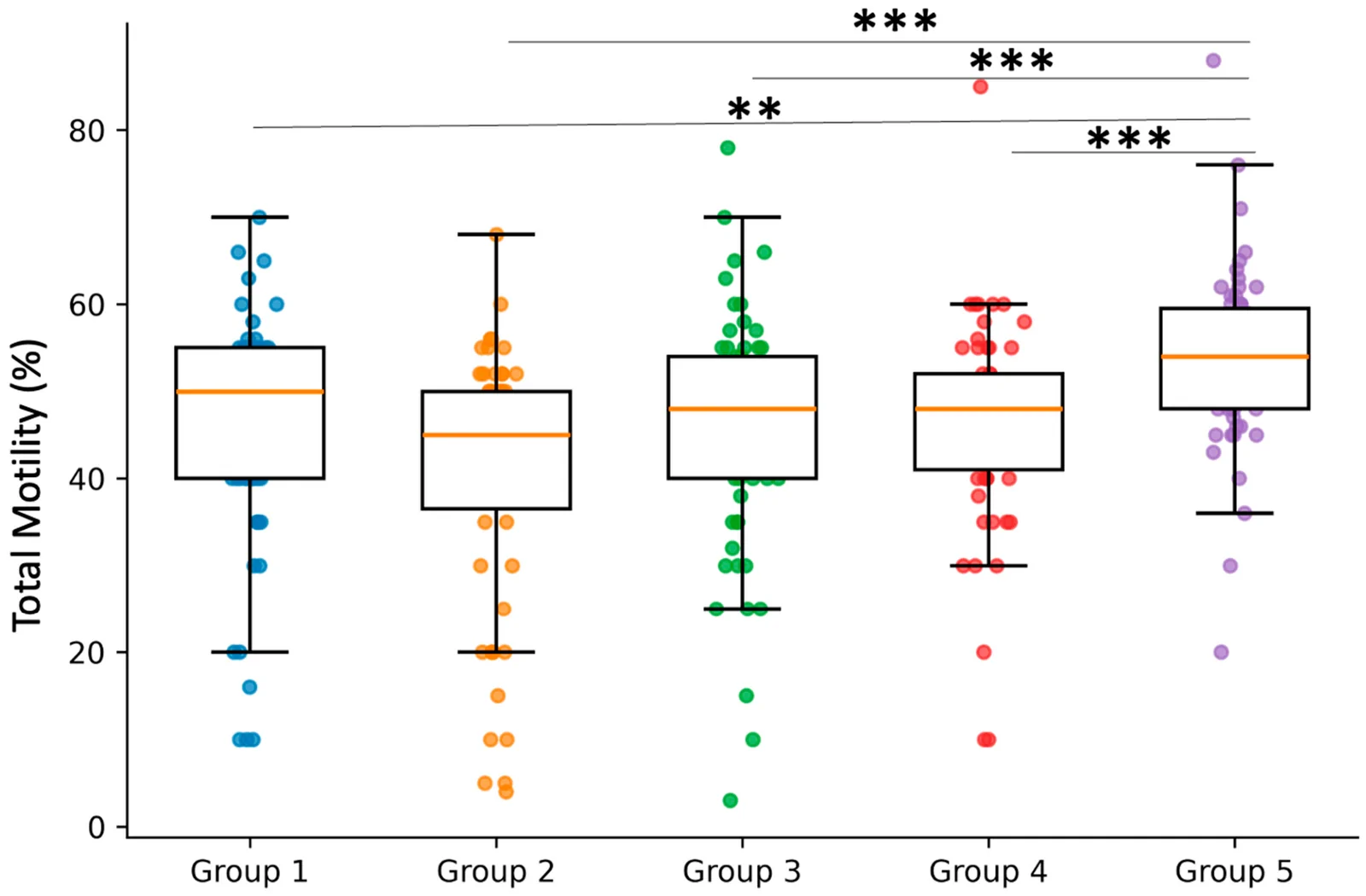
Effect of five different cryopreservation media on post-thaw total sperm motility (%). Each cryopreservation condition included paired aliquots from 63 donors (*n* = 63). Data are presented as box-and-whisker plots (median, IQR, minimum–maximum) with individual data points. Statistical significance is indicated according to Bonferroni-adjusted pairwise comparisons: *P*_adj < 0.01 (**); *P*_adj < 0.001 (***). Selected statistically significant pairwise comparisons are displayed; all adjusted pairwise *p* values are reported in Table 1.

Post-thaw total sperm motility was evaluated in all 63 donors (*n* = 63) and differed significantly among the five cryopreservation groups (Figure 1). The median total sperm motility was 50% (IQR: 40–55%) in Group 1, 45% (IQR: 36.5–50%) in Group 2, 48% (IQR: 40–54%) in Group 3, 48% (IQR: 41–52%) in Group 4, and 54% (IQR: 48–59.5%) in Group 5. Group 5 showed the highest median total motility among all groups.

**Figure 2:**
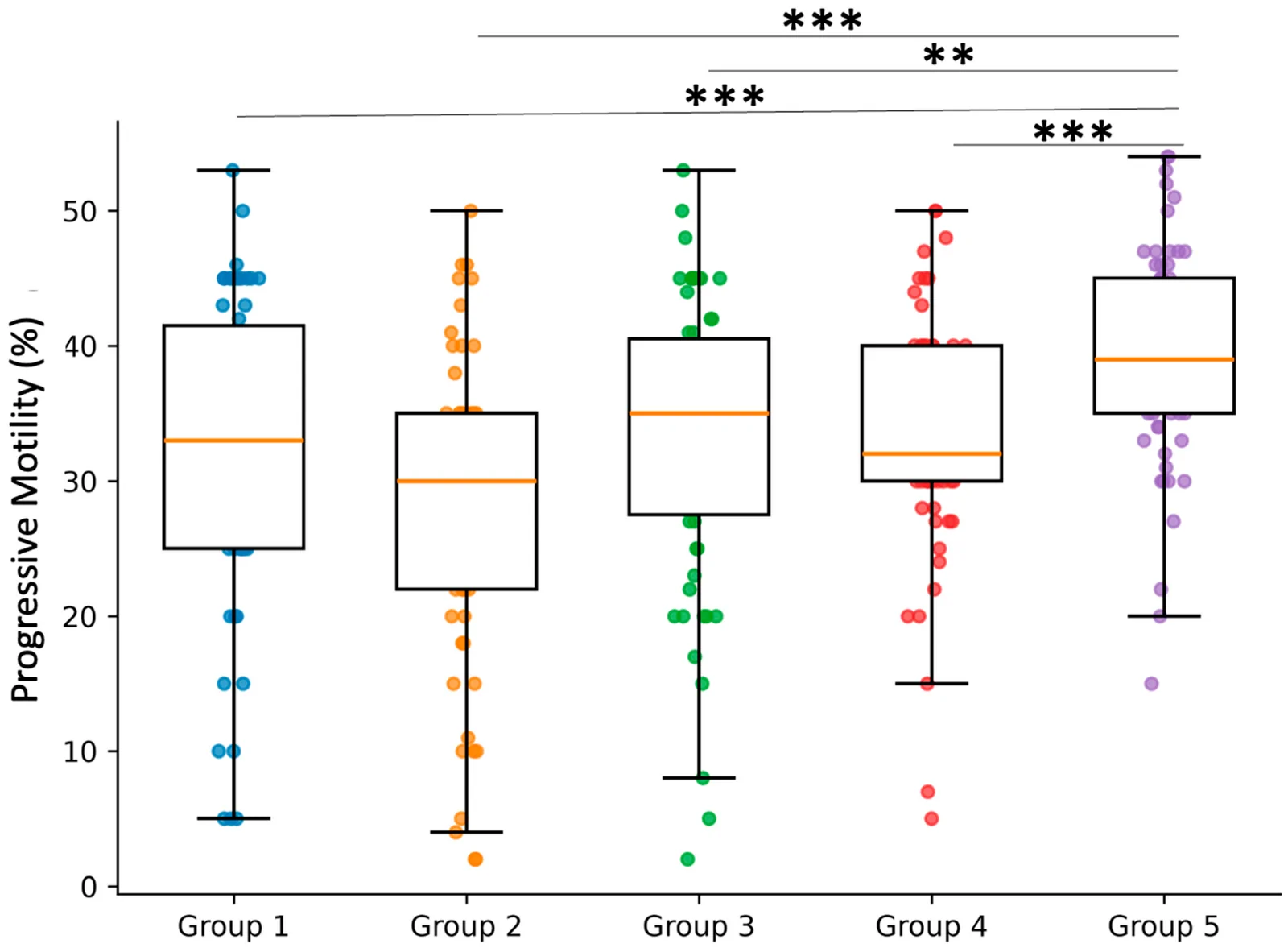
Effects of five different cryopreservation media on post-thaw progressive sperm motility (%). Each cryopreservation condition included paired aliquots from 63 donors (*n* = 63). Data are shown as box-and-whisker plots with individual data points. Statistical significance is indicated according to Bonferroni-adjusted pairwise comparisons: *P*_adj < 0.01 (**); *P*_adj < 0.001 (***). Selected statistically significant pairwise comparisons are displayed; all adjusted pairwise *p* values are reported in Table 1.

A similar pattern was observed for progressive sperm motility (Figure 2). The median progressive motility was 33% (IQR: 25–41.5%) in Group 1, 30% (IQR: 22–35%) in Group 2, 35% (IQR: 27.5–40.5%) in Group 3, 32% (IQR: 30–40%) in Group 4, and 39% (IQR: 35–45%) in Group 5. Group 5 exhibited the highest progressive motility. Accounting for the paired repeated-measures structure of the aliquots, the Friedman test demonstrated significant overall differences among the five groups for both total and progressive motility. Pairwise Wilcoxon signed-rank tests with Bonferroni correction showed significant differences in total motility between Groups 1 and 2 (*P*_adj = 0.008), Groups 1 and 5 (*P*_adj = 0.002), Groups 2 and 4 (*P*_adj = 0.004), Groups 2 and 5 (*P*_adj < 0.001), Groups 3 and 5 (*P*_adj < 0.001), and Groups 4 and 5 (*P*_adj < 0.001). For progressive motility, significant differences were observed between Groups 1 and 5 (*P*_adj < 0.001), Groups 2 and 3 (*P*_adj = 0.038), Groups 2 and 4 (*P*_adj = 0.006), Groups 2 and 5 (*P*_adj < 0.001), Groups 3 and 5 (*P*_adj = 0.003), and Groups 4 and 5 (*P*_adj < 0.001) (Table 1).

### 3.2. Intracellular ROS Levels

**Figure 3:**
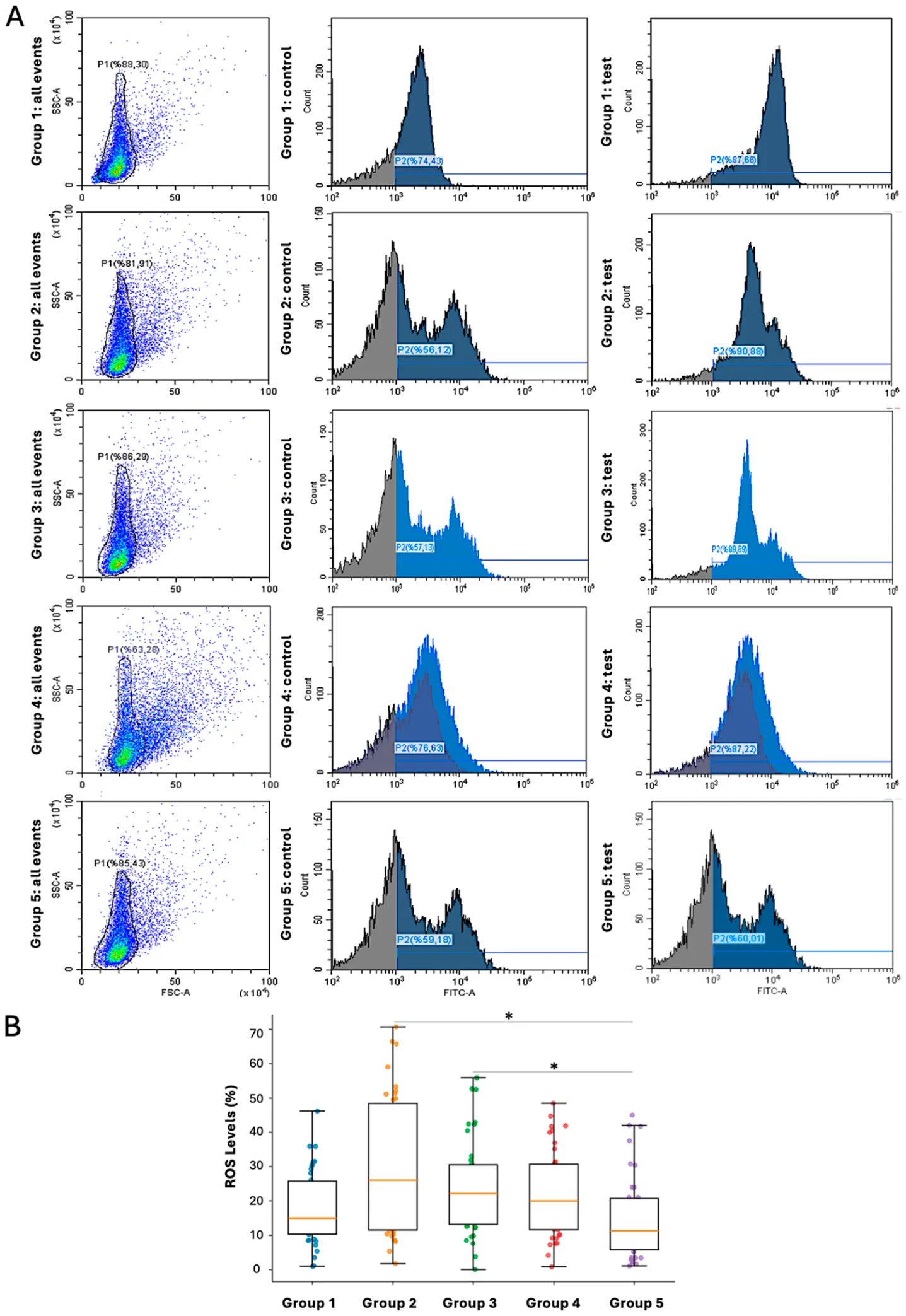
(**A**) Representative flow cytometry plots. Left panels: FSC-A and SSC-A dot plots showing the analyzed cell population (gate P1). Middle panels: ROS fluorescence (FITC-A) histograms of unstained control samples. Right panels: ROS fluorescence (FITC-A) histograms of H2DCF-DA-stained samples. The percentage of ROS-positive cells (P2 gate) is indicated on each histogram. (**B**) Quantitative comparison of intracellular ROS levels among the five groups. Each cryopreservation condition included paired aliquots from 63 donors (*n* = 63). Data are presented as box-and-whisker plots showing the median (orange line), interquartile range (box), and minimum–maximum values (whiskers), with individual data points overlaid. Differences among groups were analyzed using the non-parametric Friedman test, followed by pairwise Wilcoxon signed-rank tests with Bonferroni correction when appropriate. Statistical significance for Bonferroni-adjusted pairwise comparisons is indicated as *P*_adj < 0.05 (*).

Post-thaw intracellular ROS levels were evaluated by flow cytometry in all 63 donors (*n* = 63), and the representative flow-cytometry plots are presented in Figure 3A. The median ROS level was 14.97% (IQR: 10.30–25.74%) in Group 1, 26.05% (IQR: 11.55–48.38%) in Group 2, 22.17% (IQR: 13.17–30.55%) in Group 3, 19.98% (IQR: 11.61–30.72%) in Group 4, and 11.30% (IQR: 5.74–20.69%) in Group 5 (Figure 3B). Group 5 exhibited the lowest median intracellular ROS level among the five groups. The repeated-measures Friedman test demonstrated a significant overall difference in intracellular ROS levels among the five groups. Pairwise Wilcoxon signed-rank tests with Bonferroni correction showed significant differences between Groups 1 and 2 (*P*_adj = 0.015), Groups 2 and 5 (*P*_adj = 0.033), and Groups 3 and 5 (*P*_adj = 0.021) (Table 1).

### 3.3. Post-Thaw Sperm Viability and Apoptosis

**Figure 4:**
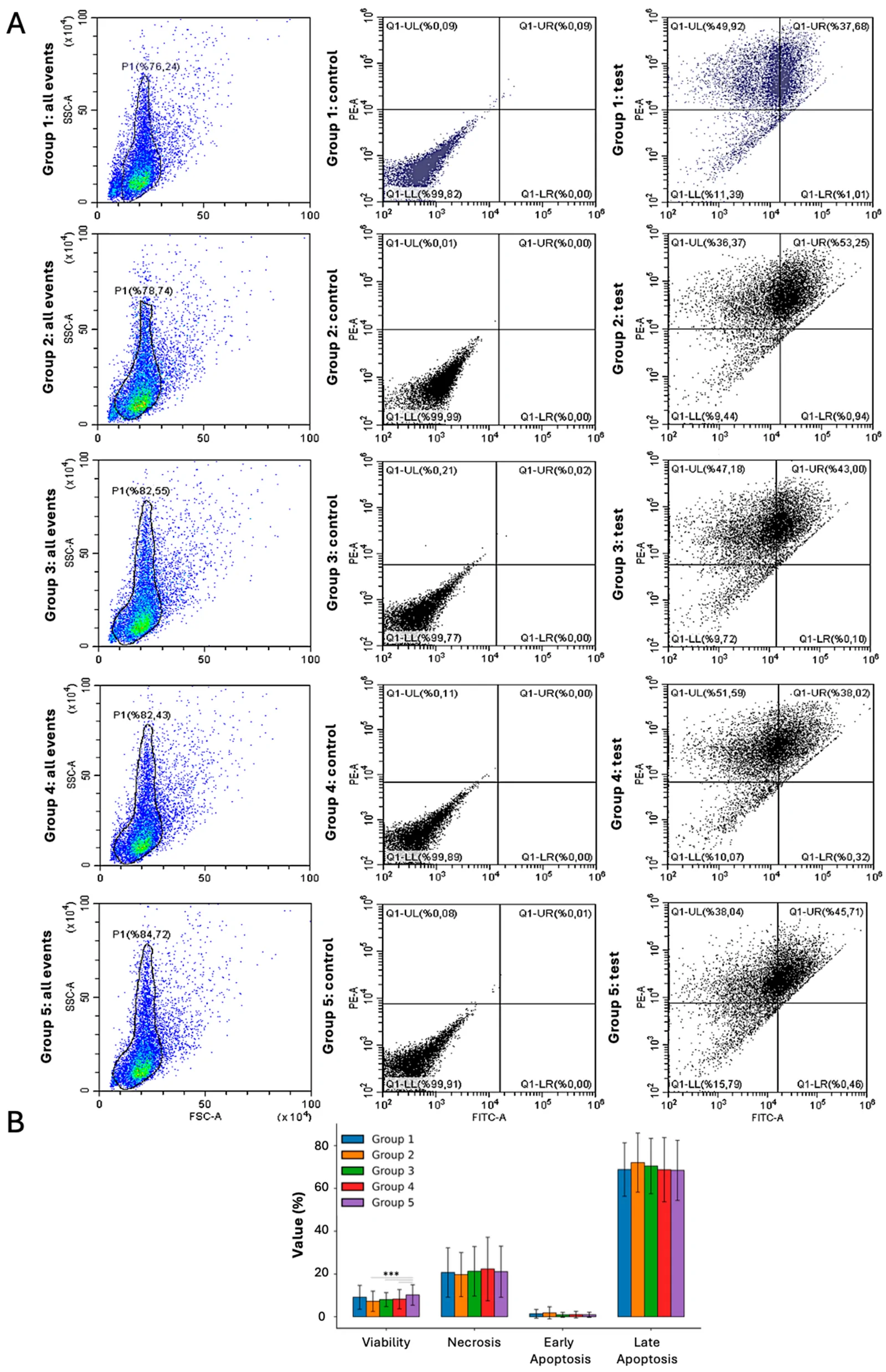
Flow cytometric evaluation of post-thaw sperm viability, necrosis, and early and late apoptosis-associated populations following cryopreservation in five different cryopreservation media. (**A**) Representative FSC-A/SSC-A plots showing the sperm gate and Annexin V-FITC/propidium iodide (PI) plots showing the staining-defined populations within the gated sperm population. (**B**) Quantitative comparison of Annexin V-negative/PI-negative (viable), necrotic, early apoptosis-associated, and late apoptosis-associated spermatozoa among the five cryopreservation media. Each cryopreservation condition included paired aliquots from 63 donors (*n* = 63). Data are presented as median and interquartile range (IQR). Differences among groups were analyzed using the non-parametric Friedman test. For Annexin V/PI-defined viability, pairwise comparisons were performed using the Wilcoxon signed-rank test with Bonferroni correction. Statistical significance for Bonferroni-adjusted pairwise comparisons is indicated as *P*_adj < 0.001 (***). No significant overall differences were observed for the necrotic, early apoptosis-associated, or late apoptosis-associated populations. FITC: fluorescein isothiocyanate; PI: propidium iodide; FSC-A: forward scatter; SSC-A: side scatter; PE-A: phycoerythrin.

Flow cytometric analysis of Annexin V-FITC/PI staining was used to evaluate post-thaw sperm viability, necrosis, and early and late apoptosis-associated populations in all 63 donors (*n* = 63) (Figure 4). The proportion of Annexin V-negative/PI-negative spermatozoa, classified as viable, was approximately 9% in Group 1, 7% in Group 2, 8% in Group 3, 8% in Group 4, and 10% in Group 5. The Friedman test showed a significant overall difference in Annexin V/PI-defined viability among the five groups (*p* < 0.001). Pairwise Wilcoxon signed-rank tests with Bonferroni correction showed significant differences between Groups 2 and 5, Groups 3 and 5, and Groups 4 and 5 (all *P*_adj < 0.001; Table 1).

Necrotic spermatozoa accounted for approximately 20–22% of the sperm population across the groups, whereas early apoptosis-associated spermatozoa represented a relatively small proportion of the population. Late apoptosis-associated spermatozoa constituted the largest fraction in all groups, with values of approximately 68–72%. No significant overall differences were detected among the five groups for necrotic (Friedman *p* = 0.324), early apoptosis-associated (*p* = 0.201), or late apoptosis-associated spermatozoa (*p* = 0.120); therefore, post-hoc pairwise comparisons were not performed for these outcomes.

### 3.4. Sperm DNA Fragmentation

**Figure 5:**
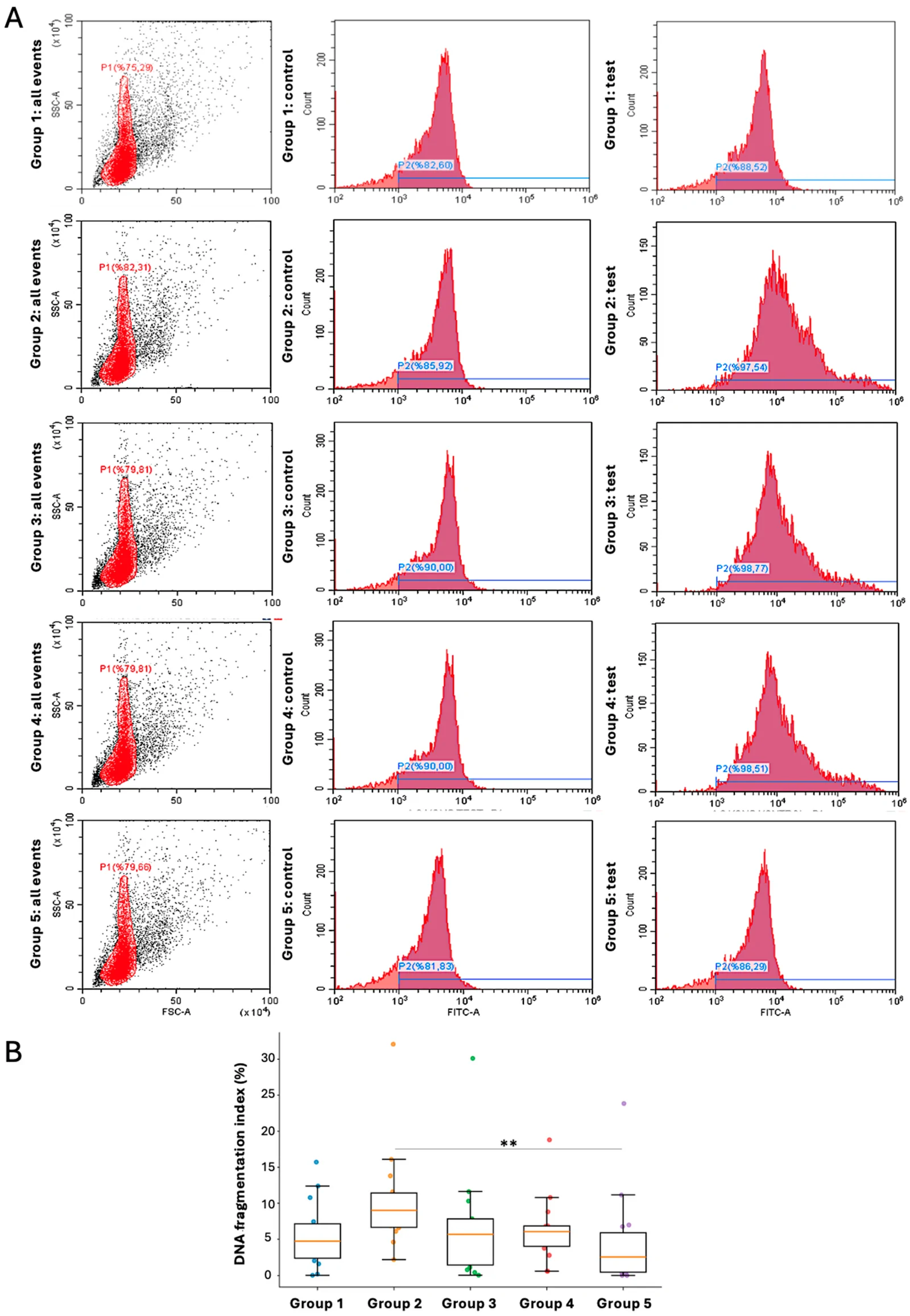
Flow cytometric evaluation of post-thaw sperm DNA fragmentation following cryopreservation in five different cryopreservation media. (**A**) Representative flow-cytometry plots of post-thaw sperm DNA fragmentation for each of the five cryopreservation groups. (**B**) Comparison of post-thaw sperm DNA fragmentation indices among the five cryopreservation media. DNA fragmentation was assessed using paired aliquots from a subset of 32 donors (*n* = 32). Data are presented as median and interquartile range (IQR). Differences among groups were analyzed using the non-parametric Friedman test, followed by pairwise Wilcoxon signed-rank tests with Bonferroni correction when appropriate. Statistical significance for Bonferroni-adjusted pairwise comparisons is indicated as *P*_adj < 0.01 (**). FSC-A: forward scatter; SSC-A: side scatter.

Post-thaw sperm DNA fragmentation was evaluated by flow cytometry in a subset of 32 participants (*n* = 32) (Figure 5). The median DNA fragmentation index was 4.73% (IQR: 2.39–7.14%) in Group 1, 9.00% (IQR: 6.66–11.43%) in Group 2, 5.69% (IQR: 1.46–7.82%) in Group 3, 6.06% (IQR: 4.01–6.84%) in Group 4, and 2.58% (IQR: 0.46–5.90%) in Group 5. Group 5 exhibited the lowest median DNA fragmentation index among the five groups. The repeated-measures Friedman test demonstrated a significant overall difference in DNA fragmentation among the five groups. Pairwise Wilcoxon signed-rank tests with Bonferroni correction showed significant differences between Groups 1 and 2 (*P*_adj = 0.012), Groups 2 and 3 (*P*_adj = 0.034), Groups 2 and 4 (*P*_adj = 0.034), and Groups 2 and 5 (*P*_adj = 0.005) (Table 1).

### 3.5. Molecular Docking

Docking was performed to explore predicted binding modes of 5-HT, L-C and coenzyme Q at sperm motility–related targets. Predicted affinities of serotonin for HTR4, SLC6A4, CPT1A, CATSPER1 and GPX4 were −6.89, −6.75, −6.83, −5.41 and −4.50 kcal/mol, respectively; corresponding values ranged from −5.10 to −3.42 kcal/mol for L-C and from −7.76 to −4.86 kcal/mol for CoQ1, and were consistent across the three runs (SD ≤ 0.14 kcal/mol; Table 2), indicating search convergence rather than pose accuracy.

**Figure 6:**
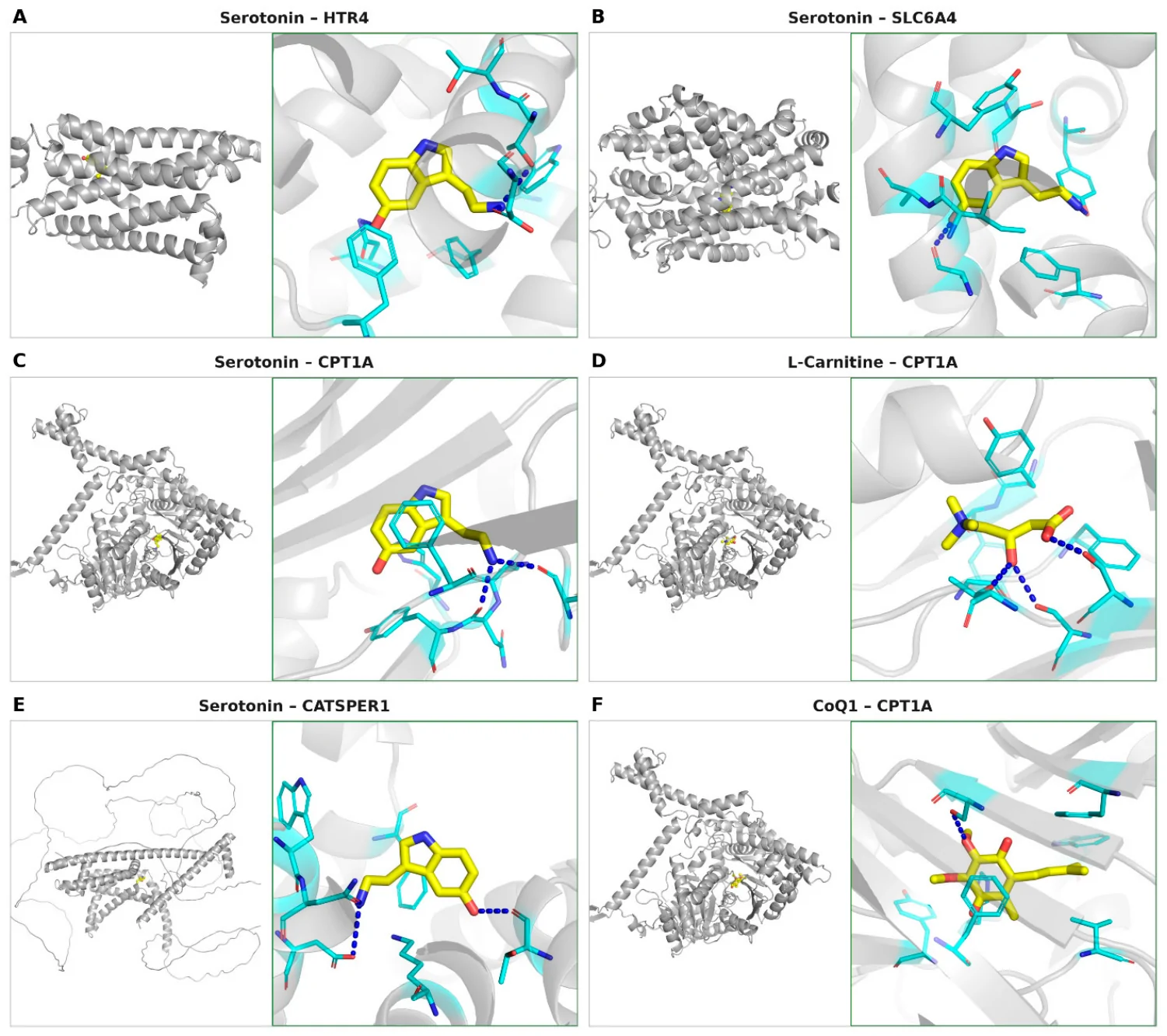
Three-dimensional docked poses of the best complexes. (**A**) serotonin–HTR4, (**B**) serotonin–SLC6A4, (**C**) serotonin–CPT1A, (**D**) L-carnitine–CPT1A, (**E**) serotonin–CATSPER1 and (**F**) CoQ1–CPT1A. For each complex the full receptor (**left**, grey cartoon) and a magnified pocket view (**right**) are shown; the ligand is drawn as yellow sticks, key interacting residues as cyan sticks, and predicted hydrogen bonds as blue dashed lines.

In the predicted HTR4 complex, serotonin formed a salt bridge with Asp99 (3.14 Å) within an aromatic cage comprising Phe185, Trp271, Phe274, Phe275 and Tyr191, with predicted hydrogen bonds to Thr103, Thr104 and Asn278 (Figure 6A). At SLC6A4, serotonin occupied the central substrate site, forming predicted hydrogen bonds with Tyr95, Ala169 and Ser438 and close aromatic contacts with Tyr95, Tyr176 and Phe341 (Figure 6B). At CPT1A, serotonin was positioned in the vicinity of the catalytic His473, although at a distance (4.09 Å) too long for a direct polar interaction, and formed aromatic contacts with Phe712 and Tyr254 together with predicted hydrogen bonds to Val250, Ser252 and Asn253 (Figure 6C). The natural substrate L-C was placed in the same region through a dense hydrogen-bond network (Ser687, Thr689, Gly710 and Ser723; all 2.7–2.9 Å), sharing Tyr254 and Phe712 with serotonin (Figure 6D), whereas CoQ1 formed only a single hydrogen bond (Ser687) despite its more favourable score of −7.76 kcal/mol (Figure 6F). The convergence of all three ligands on His473, Phe712 and Tyr254 suggests they are predicted to occupy the same pocket by different interaction types: L-C through hydrogen bonding, serotonin and CoQ1 through aromatic and hydrophobic contacts.

Blind docking of serotonin to the CATSPER1 model identified an acidic pocket in which serotonin formed a salt bridge with Asp515 (3.31 Å), predicted hydrogen bonds to Thr444, Lys498 and Asn512, and aromatic contacts with Phe565 and Trp511 (Figure 6E). In contrast, GPX4 produced the weakest scores for all three ligands (−3.42 to −4.86 kcal/mol). The predicted poses did not show consistent contacts with the GPX4 catalytic tetrad. These computational findings cannot establish or exclude ligand binding or functional effects on GPX4. This does not argue against a biological role for GPX4 in sperm oxidative protection. For all six complexes, the three-dimensional docked poses show each ligand seated within its target site and surrounded by the contacting residues (Figure 6).

Finally, a sequential-docking analysis, in which one ligand was kept fixed in the pocket and a second ligand was then docked (Δ = combined − solo), gave a positive Δ of +2.12 kcal/mol for serotonin and CoQ1 at CPT1A, with largely overlapping predicted poses, whereas L-C was placed peripherally (Supplementary Figure S1).

## 4. Discussion

Sperm cryopreservation can impair sperm function through oxidative stress, membrane damage, mitochondrial dysfunction, and DNA fragmentation. In the present study, the combined supplementation of 5-HT, L-C, and CoQ10 showed a favorable post-thaw profile, characterized by higher median total and progressive motility, lower median intracellular ROS, higher median viability, and lower median DNA fragmentation than the basal freezing medium. However, the magnitude and statistical significance of these differences varied according to the comparator and outcome. Compared with the commercial freezing medium, the combined formulation showed significantly higher total and progressive motility, whereas the differences in intracellular ROS, viability, and DNA fragmentation were not statistically significant. Thus, the findings indicate endpoint-specific improvements rather than an overall superiority of the combined formulation over the commercial freezing medium. Because aliquots from the same donor were assigned to all experimental groups, interindividual variability was minimized. These findings suggest that combining compounds with complementary biological functions may provide broader protection against cryopreservation-induced sperm damage than the basal freezing medium alone.

The reduction in ROS observed with the combined formulation is particularly relevant because oxidative stress is a major mechanism of cryoinjury and can negatively affect sperm motility, mitochondrial function, and DNA integrity [31,32]. L-carnitine may contribute by supporting mitochondrial fatty-acid metabolism and energy production, while CoQ10 functions as both a mitochondrial electron-transfer component and a lipid-soluble antioxidant. Previous studies have reported beneficial effects of L-C and CoQ10 on post-thaw sperm motility, viability, oxidative status, mitochondrial function, and DNA integrity [33,34,35,36]. The simultaneous improvement in motility and reduction in ROS in our study are therefore consistent with the protective roles previously attributed to these compounds. The formulation and concentrations were informed by our previous investigations. However, the present study did not include L-carnitine alone, CoQ10 alone, or all possible combinations of the three compounds. Consequently, the individual contributions of the components within the combined freezing formulation and any synergistic interaction cannot be established from this experimental design.

Although Annexin V/PI staining is commonly used to assess apoptosis-associated changes in spermatozoa, phosphatidylserine externalization may also reflect cryopreservation-induced membrane alterations. Therefore, these findings should be interpreted cautiously and not regarded as definitive evidence of classical apoptosis. The Annexin V-negative/PI-negative fraction was substantially lower than the proportion of motile spermatozoa. These measurements represent distinct endpoints: motility describes sperm movement, whereas Annexin V/PI classification reflects phosphatidylserine exposure and membrane permeability under the staining conditions. This distinction does not fully explain the magnitude of the discrepancy, which remains a limitation requiring cautious interpretation.

The present findings extend our previous observations using the same combination of 5-HT, L-C, and CoQ10 [7]. The previous sperm-washing study and the present cryopreservation study were conducted under separate ethics approvals and involved different participants, with no overlap of donors or biological samples. In the previous study, the formulation was used as a sperm washing medium and reduced ROS and apoptosis, whereas no significant differences were observed in motility or DNA fragmentation. In contrast, the present study evaluated the formulation after cryopreservation and thawing and showed favorable changes in several post-thaw parameters, including a significant reduction in DNA fragmentation. However, DNA fragmentation was assessed in a subset of 32 participants (*n* = 32), rather than the full cohort of 63 donors; therefore, these findings should be interpreted within this smaller sample. The differences between the two studies may reflect the distinct experimental conditions and stages of sperm processing evaluated.

The docking results generate testable hypotheses about how the three compounds may engage sperm-relevant targets. Serotonin yielded docking scores of −6.89 and −6.75 kcal/mol at its native serotonergic targets HTR4 and SLC6A4, respectively (Table 2), with predicted poses in regions associated with ligand recognition. Docking to CATSPER1 yielded a score of −5.41 kcal/mol, generating a hypothesis for future investigation of a possible link between serotonergic signalling, calcium regulation, and sperm motility [37]. L-C yielded a docking score of −4.67 kcal/mol at CPT1A, whereas CoQ1 yielded a more favorable score of −7.76 kcal/mol, with predominantly hydrophobic and aromatic contacts in the predicted pose. These scores do not establish binding specificity or experimental affinity. The predicted overlap of serotonin and CoQ1 poses at CPT1A, with L-C peripheral, suggests that these ligands may occupy a common region of the protein [38]. Whether the three compounds act on overlapping or separate molecular routes cannot be resolved by docking. However, direct ligand–protein binding assays and functional studies using target-specific inhibitors or antagonists are required to determine whether the predicted interactions occur under biological conditions and contribute to the observed sperm-protective effects.

Several limitations apply. Docking under a rigid-receptor approximation neglects protein flexibility, solvent and the membrane environment, and Vina scores correlate only weakly with experimental affinities; the analysis cannot establish the mechanism underlying the cryoprotective effects observed here. Scores are not additive across compounds and cannot apportion the effect among them. CPT1A and CATSPER1 were AlphaFold models, blind docking was required for CATSPER1, redocking validation was not performed, and CPT1A is a surrogate for the sperm-predominant isoform CPT1B. CoQ1 differs from CoQ10 in chain length and lipophilicity, so its results approximate head-group interactions only. Finally, none of the five targets was examined experimentally; confirming their involvement would require direct binding, enzyme-activity, calcium-flux or loss-of-function assays.

Overall, the combined 5-HT + L-C + CoQ10 formulation showed favorable changes in several post-thaw sperm quality parameters, including motility, intracellular ROS, viability, and DNA integrity. These findings support further investigation of this formulation in sperm cryopreservation.

## Figures and Tables

**Table 1:** Pairwise post-hoc comparisons of post-thaw sperm functional parameters using the Wilcoxon signed-rank test with Bonferroni correction following the Friedman test. Sample sizes were *n* = 63 donors for total motility, progressive motility, intracellular ROS, and Annexin V/PI-defined viability, and *n* = 32 donors for DNA fragmentation. All comparisons were based on paired aliquots from the same donors. Statistical significance: * *P*_adj < 0.05; ** *P*_adj < 0.01; *** *P*_adj < 0.001.

Comparison	Total Motility (P_adj)	Progressive Motility (P_adj)	Intracellular ROS (P_adj)	Viability (P_adj)	DNA Fragmentation (P_adj)
Group 1 vs. Group 2	0.008 **	0.059	0.015 *	0.156	0.012 *
Group 1 vs. Group 3	1.000	1.000	0.127	1.000	1.000
Group 1 vs. Group 4	1.000	1.000	1.000	1.000	0.803
Group 1 vs. Group 5	0.002 **	<0.001 ***	1.000	0.815	1.000
Group 2 vs. Group 3	0.061	0.038 *	1.000	0.127	0.034 *
Group 2 vs. Group 4	0.004 **	0.006 **	0.145	0.402	0.034 *
Group 2 vs. Group 5	<0.001 ***	<0.001 ***	0.033 *	<0.001 ***	0.005 **
Group 3 vs. Group 4	1.000	1.000	1.000	1.000	1.000
Group 3 vs. Group 5	<0.001 ***	0.003 **	0.021 *	<0.001 ***	0.942
Group 4 vs. Group 5	<0.001 ***	<0.001 ***	0.257	<0.001 ***	1.000

**Table 2:** Most favorable AutoDock Vina (version 1.2.7) docking scores (kcal/mol) for each ligand at the five targets. More negative values indicate more favorable scores within the docking model, rather than experimentally established binding affinities. GPX4 is the comparator. CoQ1 is a short-chain analogue of CoQ10. CPT1A and CATSPER1 were AlphaFold-predicted models; SLC6A4, HTR4 and GPX4 were experimentally determined structures.

Target	PDB/Model	CoQ1	Serotonin	L-Carnitine
SLC6A4	6VRH	−7.03	−6.75	−4.52
HTR4	7XT8	−7.70	−6.89	−5.10
CATSPER1	AF-Q8NEC5	−5.68	−5.41	−4.21
CPT1A	AF-P50416	−7.76	−6.83	−4.67
GPX4 (control)	6HN3	−4.86	−4.50	−3.42

## Data Availability

The data supporting the findings of this study are available from the corresponding author upon reasonable request.

## Supplementary Materials

The following supporting information can be downloaded at: https://www.mdpi.com/article/10.3390/jcm15208134/s1, Figure S1: Structural basis of competition at CPT1A.
