# Supplementary material for: Protective Effects of Serotonin, L-Carnitine, and Coenzyme Q10 on Human Sperm Cryopreservation: Functional and In Silico Evidence
Source: J Clin Med. 2026 Oct 20;15(20):8134. doi: 10.3390/jcm15208134 (PMC13705688; doi:10.3390/jcm15208134)
Supplement: Supplementary file 1 [file jcm-15-08134-s001.zip › jcm-4543046-supplementary.pdf]

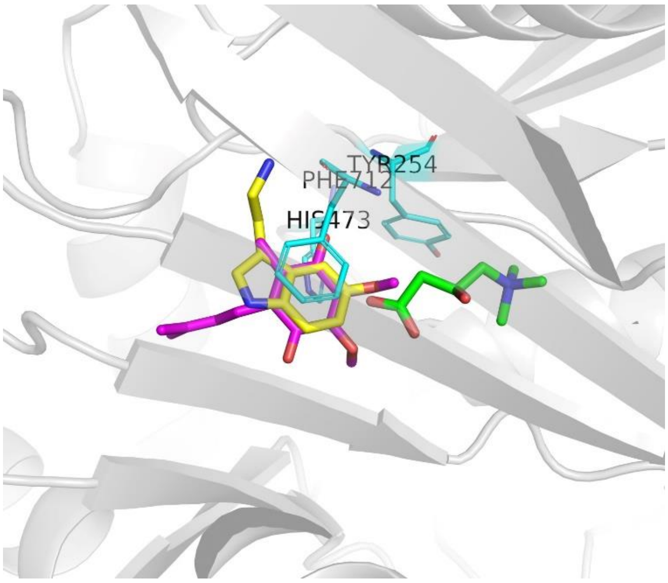

Supplementary Figure S1. Structural basis of competition at CPT1A.

Overlay of 5-HT (yellow), L-C (green) and CoQ1 (magenta) in the CPT1A catalytic pocket, sharing His473, Phe712 and Tyr254. 5-HT and CoQ1 occupy nearly identical positions; L-C binds peripherally.
